# Functional diversity outperforms taxonomic diversity in revealing short-term trampling effects

**DOI:** 10.1038/s41598-021-98372-3

**Published:** 2021-09-23

**Authors:** Wei Li, Shuqiang He, Xiping Cheng, Mingqiang Zhang

**Affiliations:** 1 School of Geography and Ecotourism, Southwest Forestry University, Kunming, 650224 Yunnan China; 2 College of Chemistry, Chemical Engineering and Environment, Minnan Normal University, Zhangzhou, 363000 Fujian China

**Keywords:** Grassland ecology, Ecology, Environmental sciences

## Abstract

Alpine grasslands harbor diverse groups of flora and fauna, provide important ecosystem functions, and yield essential ecosystem goods and services, especially for the development of nature-based tourism. However, they are experiencing increasing anthropogenic perturbations such as tourist trampling. Although negative effects of tourist trampling on alpine vegetation have been frequently reported, previous studies have focused mainly on changes in taxonomic diversity after trampling, and rarely provide a mechanistic elucidation of trampling effects from a trait-based perspective. The present study evaluates the impacts of simulated trampling on taxonomic and functional diversity of a typical alpine grassland community in Shangri-La, China using a standardized protocol. The results showed that although taxonomic diversity was not statistically significantly affected by trampling, some functional attributes responded rapidly to trampling disturbance. Specifically, functional divergence decreased with an increase in trampling intensity, and characteristics of community-weighted mean trait values changed towards shorter species with reduced leaf area and lower leaf dry matter content. Such strong shifts in functional attributes may further affect ecosystem goods and services provided by alpine grasslands. Our inclusion of functional diversity in the analysis thus adds an important caution to previous studies predominantly focusing on taxonomic diversity, and it is urgent to keep alpine grasslands well managed and ecologically coherent so that their valuable functions and services can be safeguarded.

## Introduction

Alpine grasslands represent an important ecosystem type globally, as they harbor diverse groups of flora and fauna^1^, provide important ecosystem functions such as climate change mitigation through carbon sequestration, and yield essential ecosystem goods and services (e.g., pasture for grazing livestock) that sustain human society^2^. However, they are currently experiencing increasing threats from human activities, such as widespread overgrazing and landscape conversions^3^. In recent years, as more and more visitors are attracted to alpine grasslands for their picturesque scenery, human trampling resulting from recreation adds another threat to alpine vegetation.

Human trampling can affect vegetation directly and indirectly. On the one hand, trampling causes mechanical damage to plant tissues and organs, resulting in significant changes in morphological and physiological characteristics of plants being trampled^4–6^. The growth and reproduction of plants can also be affected by trampling in an indirect way. Trampling often leads to soil compaction, which may then limit soil exploration by plant roots and cause soil oxygen deficiency. Consequently, root growth rates, seed germination rates and seedling survival rates could all be seriously reduced or impeded^7,8^.

In addition to its effect on individual plant species, trampling also affects plant communities. For example, many observational studies have reported that trampling activities are associated with changes in vegetation cover^9–11^. However, it is difficult to tell whether a dramatic reduction in vegetation cover is due to high trampling intensity or the presence of highly sensitive species^9,12^. Cole and Bayfield developed a standardized protocol for conducting trampling experiments^13^, which has been widely adopted by ecologists worldwide to test trampling effects on plant communities and search for generality across different vegetation types and environmental conditions. However, relevant trampling experiments have been largely confined to North America and Europe, and such studies mainly focus on the vulnerability of plants using measures of resistance and resilience based on changes in vegetation cover^14^.

Biodiversity is often quantified as richness (i.e., the number of species) or other taxonomy-based diversity metrics such as Simpson index and Shannon index. However, such taxonomic diversity metrics tend to have low explanatory power, and fail to provide a mechanistic understanding of disturbance effects^15,16^. In recent years, functional trait research has gained popularity as recognition has grown indicating that functional traits mediate how a species responds to and affects its environment. Trait-based diversity metrics enhance our mechanistic understanding of the impacts of external disturbances on plant communities by combining a variety of functional traits into a comprehensive measure of functional diversity changes^17–20^. However, simulated trampling studies following the standardized protocol and conducted in natural vegetation rarely adopt a trait-based perspective (but see^21,22^), and thus the capacity to improve our mechanistic understanding of trampling effects on vegetation using functional trait information has not been fully exploited. In particular, it is unclear whether traits-based diversity metrics, such as functional richness, functional evenness, functional divergence and community-weighted mean (CWM) trait values, could better reveal changes in community structure along trampling disturbance gradients than taxonomy-based diversity metrics.

Shangri-La, a county-level city in Northwest Yunnan, China, contains large areas of alpine grasslands with significant aesthetic and recreational values, which attract large numbers of national and international tourists each year. Blue Moon Valley is one of such popular tourist attraction in Shangri-La. However, the majority of grasslands in this scenic area are accessible to the public and subject to tourism trampling without appropriate protection. Here we conducted a trampling experiment following the standardized protocol developed by Cole and Bayfield^13^. Different from previous trampling experiments that focus on testing the resistance and resilience of vegetation after disturbance, we take a trait-based approach to test short-term effects of experimental trampling on alpine functional diversity, and compare the sensitivity of trait-based versus taxonomy-based diversity metrics in response to trampling disturbance.

## Results

**Figure 1:**
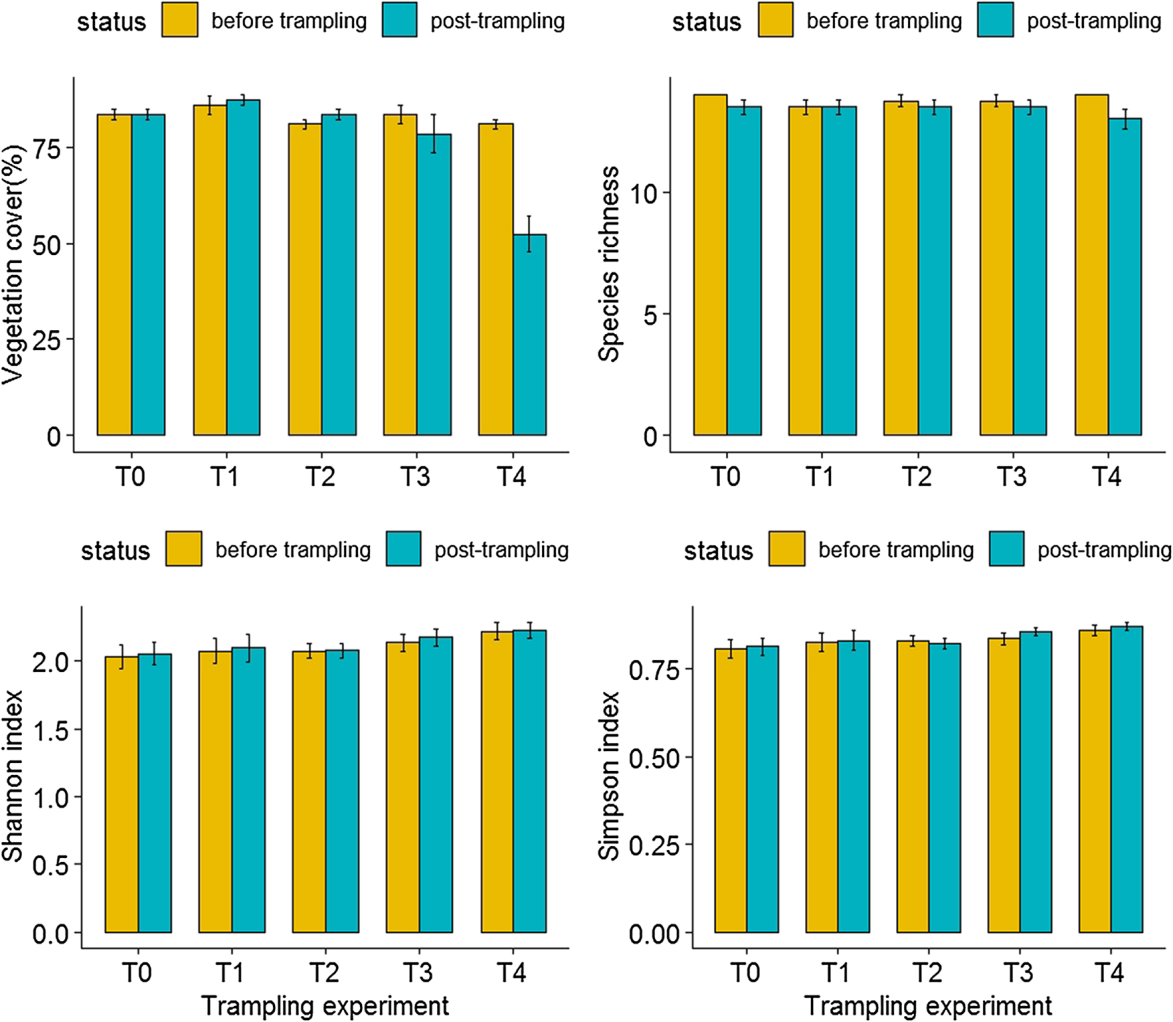
Effects of trampling disturbance on total vegetation cover and taxonomic diversity metrics of grassland communities. Species richness, Simpson index and Shannon index is used to quantify taxonomic diversity, and T_0_, T_1_, T_2_, T_3_, T_4_ and T_5_ represents experimental lanes receiving 0, 25, 75, 250 and 500 trampling passes, respectively.

**Figure 2:**
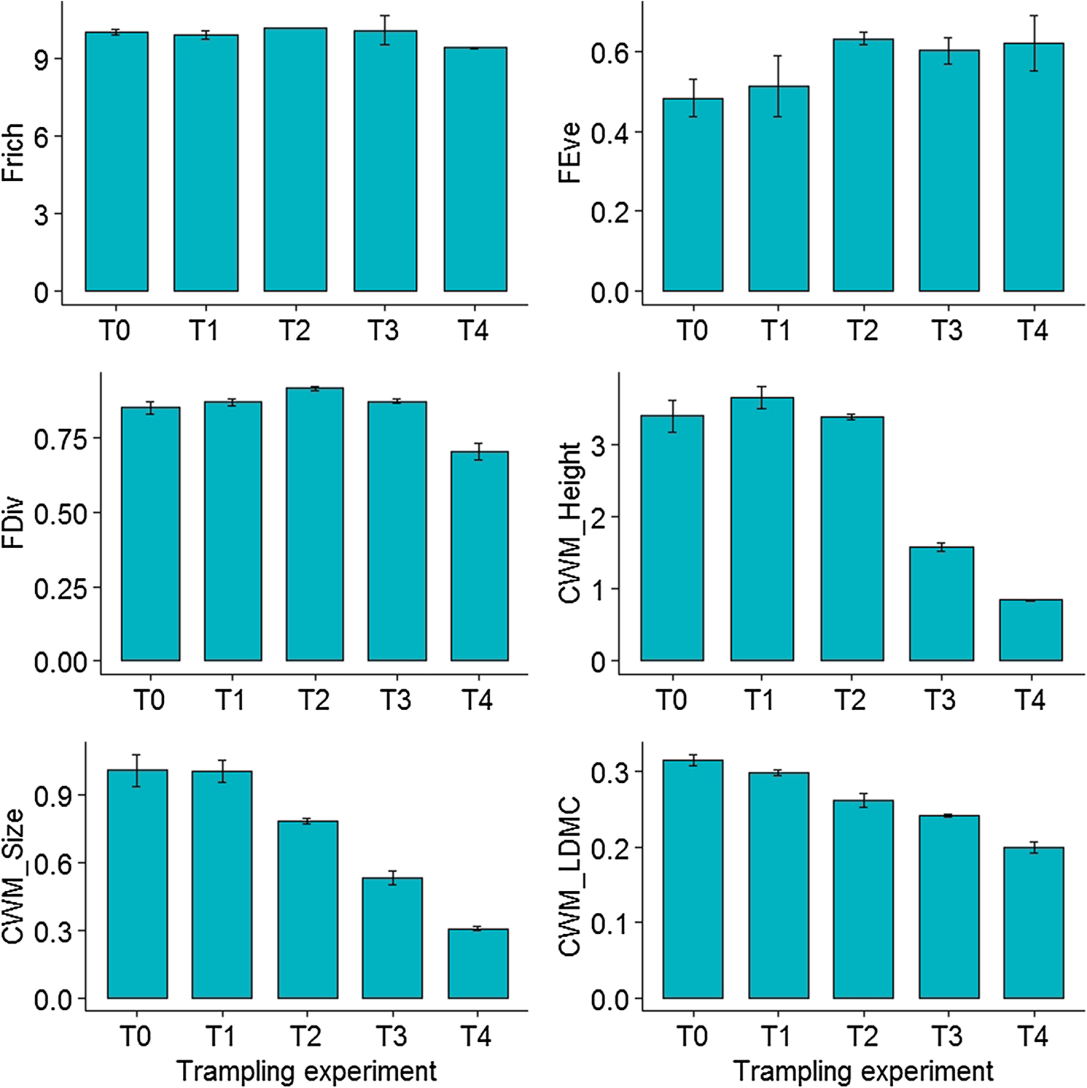
Effects of trampling disturbance on multivariate functional diversity metrics and community-weighted mean (CWM) trait values of grassland communities. Functional richness (FRich), functional evenness (FEve) and functional divergence (FDiv) is used to represent multivariate functional diversity, and CWM values of plant height (PH), leaf area (LA) and leaf dry matter content (LDMC) are quantified. T_0_, T_1_, T_2_, T_3_, T_4_ and T_5_ represents experimental lanes receiving 0, 25, 75, 250 and 500 trampling passes, respectively.

At the species level, trampling disturbance caused pronounced reductions in morphological traits such as plant height (F_4,269_ = 42.53, *p* < 0.001; Table 1) and leaf area (F_4,269_ = 15.29, *p* < 0.001; Table 2). At the community level, there was no significant difference in vegetation cover before trampling (F_4,15_ = 1.36, *p* = 0.30), but vegetation cover decreased significantly with increasing trampling intensity (F_4,15_ = 18.39, *p* < 0.001; Table 1). There was no statistically significant difference among treatment groups for species richness (F_4,15_ = 1.05, *p* = 0.42 for pre-treatment, F_4,15_ = 0.51, *p* = 0.74 for post-treatment), Simpson index (F_4,15_ = 0.89, *p* = 0.49 for pre-treatment, F_4,15_ = 1.55, *p* = 0.24 for post-treatment) and Shannon index (F_4,15_ = 0.95, *p* = 0.41 for pre-treatment, F_4,15_ = 0.98, *p* = 0.45 for post-treatment) before or after trampling experiment (Fig. 1). Similarly, neither functional richness (F_4,15_ = 1.41, *p* = 0.28) nor functional evenness (F_4,15_ = 1.61, *p* = 0.22) differed significantly among treatment groups (Fig. 2). By contrast, trampling disturbance significantly affected functional divergence (F_4,15_ = 24.59, *p* < 0.001), with treatment lanes receiving the highest level of trampling intensity (i.e., 500 passes) displayed lower functional divergence values relative to other treatment lanes (Fig. 2). Also, trampling disturbance imposed significant influence over CWM trait values of alpine vegetation (for CWM_Height, F_4,15_ = 103.7, *p* < 0.001; for CWM_Leaf area, F_4,15_ = 55.07, *p* < 0.001; for CWM_LDMC, F_4,15_ = 50.63, *p* < 0.001). With an increase in trampling intensity, the CWM traits shifted towards shorter height, reduced leaf area and lower leaf dry matter content (Fig. 2). The species included in the functional analysis represented more than 80% of the total cover within each of the five treatments (see Table S1 in Supplementary information). Decomposition of total variability in height showed that the among-block variability caused by intraspecific variability is nearly 22 times higher than that caused by species turnover (0.0345 vs 0.7616). Moreover, the effect of total trait variability is well explained by the experimental treatments as the unexplained variability is low (only 0.004 out of 0.7616 unexplained), whereas the species composition effect is explained very poorly (0.0212 out of 0.0345 unexplained; Table 3). Similarly, decomposition of total variability in leaf area showed that the among-block variability caused by intraspecific variability is nearly 19 times higher than that caused by species turnover (0.0458 vs 0.8869). Moreover, the effect of total trait variability is well explained by the experimental treatments as the unexplained variability is low (only 0.0142 out of 0.8869 unexplained), whereas the species composition effect is explained very poorly (0.0426 out of 0.0458 unexplained; Table 4).

**Table 1:** Effects of trampling disturbance on plant height (mean ± SE) of individual plant species.

Species	T0	T1	T2	T3	T4
*Ranunculus yunnanensis*	3.80 ± 0.27^a^	3.83 ± 0.38^a^	3.62 ± 0.25^a^	1.30 ± 0.23^b^	0.78 ± 0.15^b^
*Ranunculus repens*	2.55 ± 0.29^a^	2.55 ± 0.30^a^	3.00 ± 0.22^a^	1.23 ± 0.22^b^	0.88 ± 0.11^b^
*Plantago depressa*	1.38 ± 0.21^a^	1.35 ± 0.21^a^	2.10 ± 0.25^a^	1.03 ± 0.24^b^	0.60 ± 0.11^b^
*Blysmus sinocompressus*	3.03 ± 0.34^a^	3.98 ± 0.38^a^	4.30 ± 0.33^ab^	1.93 ± 0.18^c^	1.00 ± 0.09^c^
*Potentilla fulgens*	4.38 ± 0.33^a^	4.65 ± 0.40^a^	4.73 ± 0.23^a^	2.95 ± 0.26^b^	2.08 ± 0.35^b^
*Eragrostis minor*	5.20 ± 0.47^a^	5.53 ± 0.32^a^	5.00 ± 0.29^a^	2.73 ± 0.36^b^	0.90 ± 0.13^c^
*Poa annua*	3.25 ± 0.40^a^	3.50 ± 0.59^a^	2.95 ± 0.13^a^	1.68 ± 0.32^b^	1.25 ± 0.17^b^
*Sonchus oleraceus*	3.70 ± 0.44^ab^	4.15 ± 0.63^a^	2.88 ± 0.33^bc^	2.50 ± 0.24^c^	0.95 ± 0.16^d^
*Kobresia humilis*	5.45 ± 0.45^a^	4.90 ± 0.27^a^	3.33 ± 0.22^b^	1.50 ± 0.26^c^	0.95 ± 0.06^c^
*Tibetia yunnanensis*	1.63 ± 0.23^a^	1.53 ± 0.13^a^	1.15 ± 0.12^a^	0.45 ± 0.06^b^	0.28 ± 0.05^b^

T0, T1, T2, T3, T4 and T5 represents experimental lanes receiving 0, 25, 75, 250 and 500 trampling passes, respectively. Different letters indicate statistically significant difference at *p* < 0.05.

**Table 2:** Effects of trampling disturbance on leaf area (mean ± SE) of individual plant species.

Species	T0	T1	T2	T3	T4
*Ranunculus yunnanensis*	0.36 ± 0.04^a^	0.35 ± 0.04^a^	0.31 ± 0.05^a^	0.15 ± 0.02^b^	0.10 ± 0.01^b^
*Ranunculus repens*	0.27 ± 0.04^a^	0.35 ± 0.03^a^	0.29 ± 0.03^a^	0.11 ± 0.02^b^	0.08 ± 0.01^b^
*Plantago depressa*	2.83 ± 0.27^a^	2.41 ± 0.21^a^	2.07 ± 0.17^ab^	1.38 ± 0.22^b^	0.84 ± 0.08^bc^
*Blysmus sinocompressus*	0.88 ± 0.05^a^	1.03 ± 0.08^a^	0.99 ± 0.06^a^	0.72 ± 0.06^ab^	0.33 ± 0.05^b^
*Potentilla fulgens*	1.62 ± 0.14^a^	1.44 ± 0.05^a^	1.31 ± 0.05^a^	0.89 ± 0.05^b^	0.69 ± 0.09^b^
*Eragrostis minor*	1.74 ± 0.21^a^	1.79 ± 0.16^a^	1.25 ± 0.03^a^	0.92 ± 0.04^b^	0.32 ± 0.03^c^
*Poa annua*	0.57 ± 0.06^ab^	0.70 ± 0.07^a^	0.68 ± 0.03^a^	0.39 ± 0.05^b^	0.15 ± 0.03^c^
*Sonchus oleraceus*	0.65 ± 0.05^a^	0.66 ± 0.04^a^	0.50 ± 0.02^a^	0.31 ± 0.04^b^	0.10 ± 0.01^c^
*Kobresia humilis*	0.71 ± 0.07^a^	0.70 ± 0.05^a^	0.35 ± 0.03^b^	0.19 ± 0.03^c^	0.10 ± 0.02^c^
*Tibetia yunnanensis*	0.16 ± 0.03^a^	0.16 ± 0.02^a^	0.10 ± 0.01^a^	0.04 ± 0.01^b^	0.04 ± 0.01^b^

T0, T1, T2, T3, T4 and T5 represents experimental lanes receiving 0, 25, 75, 250 and 500 trampling passes, respectively. Different letters indicate statistically significant difference at *p* < 0.05.

**Table 3:** Summary tables of the decomposition of total variability in plant height.

(A)				
	DF	SS	MS	
**Fixed**				
Block	3	0.1705	0.0568	
Treatment	4	0.3564	0.0891	
Block x Treatment	12	0.3992	0.0333	
Total	19	0.9261	0.1792	
**Specific**				
Block	3	0.321	0.107	
Treatment	4	25.891	6.473	
Block x Treatment	12	0.612	0.051	
Total	19	26.824	6.631	
**Intraspecific variability**				
Block	3	0.036	0.012	
Treatment	4	20.322	5.08	
Block x Treatment	12	0.072	0.006	
Total	19	20.43	5.098	

(B)				
	Turnover	Intraspecific variability	Covariation	Total = specific average
Treatment	0.3564	20.3217	5.2131	25.8912
Error	0.5696	0.1080	0.2558	0.9335
Total	0.9260	20.4296	5.4690	26.8247

(C)				
	Turnover	Intraspecific variability	Covariation	Total = specific average
Treatment	0.0133	0.7576	0.1943	0.9652
Error	0.0212	0.0040	0.0095	0.0348
Total	0.0345	0.7616	0.2039	1.000

(A) Fixed averages, specific averages and intraspecific variability effect analyzed separately. (B) Variability of individual component of height variation, and their parts explained by experimental treatments. Covariation is obtained by subtracting the first two columns from the last. C) Proportions of variability of individual components, and their parts explained by experimental treatments. SS (sum of squares) correspond to the amount of variability. Significant *p*-values (*p* < 0.05) are in bold.

**Table 4:** Summary tables of the decomposition of total variability in leaf area.

(A)				
	DF	SS	MS	
**Fixed**				
Block	3	0.0043	0.0014	
Treatment	4	0.0050	0.0013	
Block x Treatment	12	0.0631	0.0053	
Total	19	0.0724	0.0080	
**Specific**				
Block	3	0.0236	0.0079	
Treatment	4	1.4786	0.3697	
Block x Treatment	12	0.0787	0.0066	
Total	19	1.5809	0.3842	
**Intraspecific variability**				
Block	3	0.0097	0.0032	
Treatment	4	1.3798	0.3449	
Block x Treatment	12	0.0127	0.0011	
Total	19	1.4022	0.3492	

(B)				
	Turnover	Intraspecific variability	Covariation	Total = specific average
Treatment	0.0050	1.3798	0.0938	1.4786
Error	0.0673	0.0224	0.0126	0.1023
Total	0.0724	1.4022	0.1064	1.5809

(C)				
	Turnover	Intraspecific variability	Covariation	Total = specific average
Treatment	0.0032	0.8728	0.0594	0.9353
Error	0.0426	0.0142	0.0080	0.0647
Total	0.0458	0.8869	0.0673	1.000

(A) Fixed averages, specific averages and intraspecific variability effect analyzed separately. (B) Variability of individual component of height variation, and their parts explained by experimental treatments. Covariation is obtained by subtracting the first two columns from the last. (C) Proportions of variability of individual components, and their parts explained by experimental treatments. SS (sum of squares) correspond to the amount of variability. Significant *p*-values (*p* < 0.05) are in bold.

## Discussion

The present study evaluates the impacts of simulated trampling on the taxonomic and functional diversity of a typical alpine grassland community in Shangri-La, China following a standardized experimental protocol. Previous studies suggest that functional diversity metrics are more sensitive to external disturbances, and thus are more able to discern disturbance impacts than are basic measures of taxonomic diversity metrics^15,23,24^. Our results corroborate these views as no significant effects of trampling intensity on all three taxonomic diversity metrics were observed. This lack of trampling effect was probably due to relatively low soil moisture of our research site. In fact, other studies also report that plants grow in dry soils are generally more resistant to external disturbances than plants growing in wet soils^25–27^. Another explanation is that alpine grasslands in this area experience a history of frequent disturbances, and thus are adapted to regular and intense disturbances. By contrast, several measures of functional diversity, such as functional divergence and the average plant trait values in the community (the community-weighted mean, CWM), did respond significantly to trampling disturbance. Specifically, at high disturbance intensity, the alpine grassland community had shortened height, smaller leaf area and lower leaf dry matter content when compared to grassland community receiving lower levels of trampling stress. Moreover, functional divergence decreased with an increase in trampling intensity. This decrease in functional divergence suggests an increase in functional redundancy, or, in other words, there is likely to be a functionally equivalent species in the grassland community if another species is lost^28,29^, and that is probably the reason why both functional richness and evenness is independent of trampling disturbance. However, lower functional divergence also means niche homogenization among species^24,30^. As species become more functionally similar, higher competitive pressure may follow, with the potential for the long-term erosion of biodiversity and ecosystem functions.

Historically, ecologists have debated whether taxonomic or functional diversity metrics are more sensitive in the face of external disturbances, and thus are more capable of capturing the response signals of biological communities. However, in recent years, they generally agree that functional diversity metrics complement taxonomic diversity metrics, and people could have a better understanding of external disturbances on biological communities through complementary analyses of both taxonomic and functional diversity metrics^17,31–33^. Also, conservation strategies can be improved by considering the consequences of anthropogenic perturbations for both taxonomic and functional diversity^23,34^. Our results further suggest that we also need to consider different aspects of taxonomic and functional diversity metrics. In our case, trampling disturbance did not alter some measures of functional diversity such as functional richness and functional evenness, but did change others such as functional divergence and community-weighted mean trait values.

Ecosystem functioning can be inferred through functional diversity metrics^18,31,33^. For the present study, alpine grassland communities that experienced heavy trampling pressure may have reduced capacities for carbon storage (e.g., reduced leaf area is associated with lower photosynthetic capacity), which could further negatively affect biomass production and the flow of ecosystem services from alpine grasslands to human societies. Meanwhile, trampling disturbance might release competitive pressure over short time scales by enhancing light accessibility aboveground or nutrient availability belowground, which is beneficial for the regrowth of alpine vegetation after trampling disturbance. However, lower leaf dry matter content also means alpine plants might have put low investment in defense, and thus they are highly vulnerable to herbivory during the regrowth phase.

Our results showed that the effect of intraspecific trait variability was dominant, and trait plasticity might have played an important role in plant response to trampling disturbance, as higher trait plasticity can help decrease vulnerability of species to disturbance. Therefore, neglecting intraspecific trait variability may lead to underestimation of community trait composition response to external disturbance. However, since the effect of species composition could be more marked for some other traits or along some other environmental gradients^42^, in order to have a more complete understanding of the responses of species trait averages to environment, both fixed trait averages and specific trait averages should be quantified and then compared. It is worth noting that it is not realistically possible to sample every species for every trait. In order to capture the effects of key ecosystem processes, a general rule of thumb is that species which collectively make up for at least 80% of the total cover for each plot should be selected and sampled^43,44^.

One caveat of the present study is that it only tests the effect of a single disturbance event. Arid grasslands of our study site are subject to multiple types of disturbances such as uncontrolled grazing, turf removal and climate change. However, we had to compromise our experimental design to ensure that the observed patterns are assigned to trampling disturbance rather than other factors, to capture the transient response signals of alpine vegetation, and to elucidate the short-term effects of experimental trampling on functional traits and diversity of alpine communities. However, repeated trampling disturbances, rather than single disturbance event, might impose more threats to alpine vegetation, and this is especially true when the recovery capability of alpine vegetation is undermined in the face of persistent anthropogenic disturbance. Therefore, further study should address whether alpine vegetation follows different development and recovery trajectories under the influence of single versus repeated trampling disturbance.

Overall, our study clearly showed that high-altitude alpine vegetation exhibited substantial changes in functional divergence and all single CWM trait values, such as CWM of plant height, leaf area and leaf dry matter content, in response to trampling disturbance. Specifically, functional divergence decreased with an increase in trampling intensity, and characteristics of community-weighted mean trait values changed towards those with shorter height, reduced leaf area and lower leaf dry matter content, and such strong shifts in functional attributes may further affect ecosystem goods and services provided by alpine grasslands. By contrast, we found no differences in three taxonomic diversity indices (e.g., species richness, Simpson’ index and Shannon’s index), as well as two multivariate functional diversity indices (e.g., functional richness and functional evenness) along trampling intensity. Our results underline the need to consider not only taxonomic and functional traits, but also measures of different aspects of taxonomic and functional diversity metrics, when describing the impacts of anthropogenic perturbations on community structure. We suggest that a combination of taxonomic approaches and trait-based approaches may help us better understand the impacts of multiple stressors on global alpine grasslands, including but not limited to human trampling, agricultural land conversion, grazing pressure and climate change.

## Methods

The experimental site is located at an elevation of 3270 m in Blue Moon Valley scenic area, Shangri-La, China, 27°48'N, 99°39'E, which experiences typical plateau climate. Average annual precipitation is 606 mm, and average monthly temperature ranges from -3.7 °C in January to 13.2 °C in July^35^. In our experimental site, alpine grasslands are generally distributed in homogeneous landscapes with similar soil nutrients and moisture conditions. The experimental design followed the standardized protocol: Four replicate blocks of trampling lanes were established, and each block consisted of 5 treatment lanes. Each lane was 0.5 m wide and 2 m long, with a 0.5 m buffer zone between lanes. Treatments were randomly assigned to these lanes, among which one lane was the control lane and received no trampling, and the other four lanes received different intensities of trampling (25, 75, 250, 500 trampling passes, with a completion of 2-m walk by a research participant as one pass). As the weight of the five research participants varied, each of them completed 5, 15, 50, and 100 passes, respectively, corresponding to treatment lanes assigned to 25, 75, 250 and 500 passes. The use of plants or plant parts in the present study complies with international, national and/or institutional guidelines, and we have permissions to collect plant materials from local Environmental Protection Bureaus. The voucher specimens are stored in Southwest Forestry University Botanic Garden herbarium, with a policy of giving bona fide researchers access to deposited specimens, and that they are scrupulously conserved. The voucher specimens were identified by Professor Fan Du and Mrs. Rui Tan.

The trampling treatment was applied on a typical sunny summer day, as alpine grasslands in Shangri-La typically grow vigorously in the summer season. Although species composition might vary as some species with small population size are distributed sporadically, we only took measurement of plants that occurred in at least three replicate lanes. Initial measurements were taken immediately before trampling. We identified plants to species level and then visually estimated the percent cover of each species as well as total vegetation cover. Post-trampling measurements were taken 15 days after trampling disturbance, with the abundance and percent cover of each species, as well as the total cover measured or estimated.

To have a clear understanding of post-disturbance responses of alpine grasslands from a functional trait perspective, we measured plant height, leaf area and leaf dry matter content following the standardized protocols detailed by Garnier^36^ and Cornelissen et al.^37^ These three functional traits were chosen because they are relatively independent, and they reflect the high sensitivity (e.g., plant height and leaf area) or resistance (e.g., leaf dry matter content) of plants to physical disturbance. Because laboratory measurement of functional traits required the removal of plants from each treatment lane, to minimize the impacts of other types of disturbance other than simulated trampling, functional characteristics of vegetation communities were investigated only once 15 days later after the completion of trampling treatment.

Plant height was measured directly using a ruler, and four individuals of each species were used to measure plant height. We were very careful with our selection of sampled leaves. Only young, intact leaves without the appearance of mechanical damage were chosen (produced following the trampling treatment), as the traits of such young leaves may reflect the capacity of alpine plants for acclimation in response to trampling disturbance. 3–5 intact, young but fully expanded leaves of each species were collected to measure leaf area and leaf dry matter content. To avoid dehydration and ensure in the field, these collected leaves were wrapped into damp paper towels and sealed in labeled plastic bags. Such leaf samples were then stored in a cooler until further processing in the laboratory. Fresh leaves were rehydrated for 6 h, then dried with tissue paper to remove any surface water, and weighted immediately to determine their staturated fresh mass. These leaves were then oven-dried at 60 °C for at least 2 days, and their dry mass was measured immediately after being taken from the oven to avoid absorption of moisture from the air. Leaf area was determined using CanoScan LiDE 120, and digital images of scanned leaves were analyzed using ImageJ. Leaf dry matter content was quantified as the leaf dry matter per unit leaf fresh mass. Because plant species from experimental lanes receiving high trampling intensity were with lower abundance, smaller leaves and a limited number of intact leaves when compared to their counterparts from other treatment lanes, to measure leaf dry matter content in a consistent manner, leaves of the same species from the same treatment lane were pooled across replicate blocks.

For each treatment lane, taxonomic and functional diversity metrics of alpine vegetation were quantified. Specifically, taxonomy-based diversity metrics include species richness, Simpson index and Shannon index. For functional trait-based diversity metrics, three multivariate functional indices, including functional richness, functional evenness and functional divergence, were used to collectively quantify the range, distribution and relative abundance of functional traits within a community^38,39^. Also, the average of trait values in a community was weighted by the relative abundance of each species to quantify community-weighted mean (CWM) for each functional trait^40^. Since trait changes could be caused by changes in species composition, intraspecific trait variability, or a combination of these two effects^41,42^, we further assessed their relative contribution to community aggregated averages of plant height and leaf area, as these two traits were measured for sampled species at different trampling levels for each replicate block, following the decomposition method developed by Lepš and colleagues. Fixed trait values (Fixed average) were quantified as single mean trait values per species averaged across all treatments, and thus neglected the extent of intraspecific trait variability across treatments. By contrast, specific trait values (Specific average) were quantified as trait values per species measured under different treatment conditions. By doing so, trait variability per species is allowed to vary across the experimental treatments, which is caused by changes in both species composition (turnover) and intraspecific trait variability. Lastly, intraspecific trait variability (ITV) is calculated as ITV = Specific average—Fixed average. The complete analysis was performed following R code provided by Lepš and colleagues^42^.

Differences in taxonomic and functional diversity indices among experimental groups were tested using generalized linear mixed models with block included as a random effect, and multiple comparisons were performed using Tukey’s HSD test. To correct for variance heterogeneity and provide heteroscedasticity-consistent estimations of the covariance matrix, a sandwich estimator was applied^45^. All statistical analyses were conducted in the R statistics 3.4.0 platform (R Core Development Team 2017). Vegan package^46^ and FD package^47^ were used to calculate taxonomic and functional diversity metrics, respectively.

## Supplementary Information

Supplementary Information.
