# Supplementary material for: Functional diversity outperforms taxonomic diversity in revealing short-term trampling effects
Source: Sci Rep. 2021 Sep 23;11:18889. doi: 10.1038/s41598-021-98372-3 (PMC8460817; doi:10.1038/s41598-021-98372-3)
Supplement: Supplementary file 1 — Supplementary Information. [file 41598_2021_98372_MOESM1_ESM.docx]

**Table S1 Average cover of each plant species, the sum of cover for individual species, and 80% of the total cover within each of the five treatments after trampling.** T0, T1, T2, T3, T4 and T5 represents experimental lanes receiving 0, 25, 75, 250 and 500 trampling passes, respectively.

| Species cover (%) | T0 | T1 | T2 | T3 | T4 |
| --- | --- | --- | --- | --- | --- |
| *Ranunculus yunnanensis* | 2.63±0.47 | 2.00±0.46 | 2.13±0.52 | 1.75±0.48 | 2.13±0.43 |
| *Ranunculus repens* | 13.75±2.39 | 13.75±1.25 | 11.25±2.39 | 6.25±1.25 | 3.38±0.55 |
| *Plantago depressa* | 2.75±0.85 | 2.63±1.18 | 1.63±0.47 | 2.50±0.29 | 2.75±0.48 |
| *Blysmus* *sinocompressus* | 4.75±0.63 | 3.25±0.48 | 3.50±0.65 | 2.25±0.63 | 1.63±0.47 |
| *Potentilla fulgens* | 23.75±6.25 | 31.25±6.57 | 18.75±4.27 | 15.00±3.54 | 10.00±3.54 |
| *Eragrostis minor* | 41.25±7.18 | 41.25±8.26 | 42.5±1.44 | 27.5±4.33 | 17.5±1.44 |
| *Poa annua* | 20.00±2.04 | 20.25±1.65 | 18.25±1.18 | 17.75±1.03 | 11.75±1.18 |
| *Sonchus oleraceus* | 2.50±0.65 | 3.50±1.04 | 3.75±0.63 | 1.13±0.13 | 1.50±0.35 |
| *Kobresia humilis* | 2.13±0.59 | 2.38±0.63 | 2.38±0.52 | 1.50±0.61 | 0.38±0.13 |
| *Tibetia yunnanensis* | 23.75±2.39 | 23.75±1.25 | 26.25±1.25 | 18.75±3.15 | 7.50±1.44 |
| Sum of vegetation cover for individual species (%) | 137.25±10.23 | 144.00±10.38 | 130.38±4.42 | 94.38±9.80 | 58.50±4.38 |
| 80% of total vegetation cover (%) | 67.00±1.00 | 70.00±1.15 | 67.00±1.25 | 63.00±3.15 | 42.00±3.82 |
